# A Case of Early Infantile Epileptic Encephalopathy Due to KCNQ2 Gene Mutation Presenting With Episodes of Hiccups

**DOI:** 10.7759/cureus.33164

**Published:** 2022-12-31

**Authors:** Serdar Alan, Sevde Nur Vural, Didem Aliefendioglu, Nesrin Senbil

**Affiliations:** 1 Department of Pediatrics, Kirikkale University, Faculty of Medicine, Kirikkale, TUR

**Keywords:** newborn, seizure, kcnq2, gene mutation, neonatal epileptic encephalopathy

## Abstract

Neonatal epilepsy syndromes are responsible for only 15% of the cases of neonatal seizure. An underlying genetic disorder can be detected in approximately 42% of this subgroup. KCNQ2 gene-associated epilepsies are very rare and more common presentations are self-limited familial neonatal epilepsy (SLFNE) and early infantile epileptic encephalopathies (EIEE). The most common initial seizure semiologies are tonic seizures with or without autonomic symptoms in EIEE resulting from KCNQ2 gene mutation. It is characterized by early neonatal onset seizures with suppression burst pattern on electroencephalogram and typically results in severe developmental delay. Therapeutic options for infants with KCNQ2-related EIEE are limited and there is no consensus about it in the literature. Herein, the neonate with EIEE with unexpected episodes of hiccups due to novel mutation of the KCNQ2 gene, which was reported second time, was presented and antiepileptic treatment strategies were discussed in the light of current literature.

## Introduction

The first four weeks of life are the riskiest period in childhood for epileptic seizures [1]. In addition, neonatal seizures are very important as they may be associated with poor neurodevelopmental outcomes. While the incidence of neonatal seizures is approximately 1-5/1000 live births in community-based studies, it is 8.6/1000 in studies conducted in neonatal intensive care units (NICU) [2]. The most common cause of neonatal seizures is still hypoxic-ischemic encephalopathy (HIE) in term infants, while intraventricular hemorrhage in preterm infants [2]. Neonatal epilepsy syndromes are responsible for only 15% of the cases of neonatal seizure, and an underlying genetic disorder can be detected in approximately 42% of this subgroup [3].

The KCNQ2 gene, which regulates voltage-gated potassium (K) channels, was first described in 1998 [4]. The initial reports suggested that the KCNQ2 gene was associated with self-limited familial neonatal epilepsy (SLFNE) [5,6], and later it was reported that the KCNQ2 was also associated with early infantile epileptic encephalopathies (EIEE) [7,8]. Although the same gene locus is responsible for these two disorders, SLFNE and EIEE have opposite clinical outcomes [9]. EIEE is characterized by early neonatal onset persistent seizures with suppression burst patterns on electroencephalogram (EEG) and typically results in severe developmental delay [9,10].

Herein, a case of EIEE with unexpected persistent episodes of hiccups due to mutation of the KCNQ2 gene was presented, and anti-seizure treatment strategies were discussed for this rare neurologic disorder in the light of the current literature.

## Case presentation

**Figure 1:**
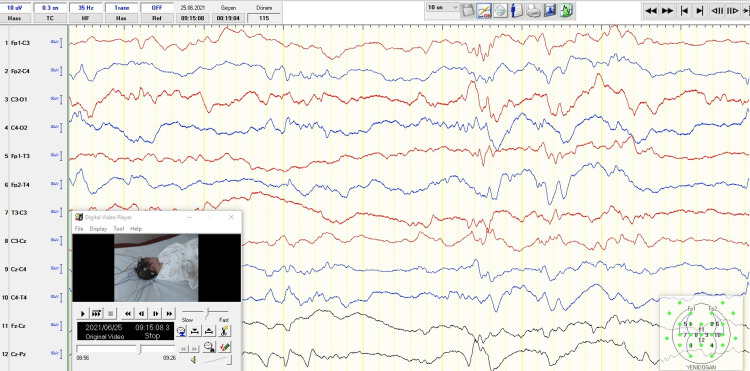
Burst suppression periods and multifocal active sharp waves were seen on EEG with newborn montage during quiet sleep at 11 days of age.

A 2770 g newborn female with a gestational age of 39 2/7 weeks was born by cesarean section to a 26-year-old gravida-1 para-0 mother in the setting of poor progression of labor after an uneventful pregnancy. APGAR scores at the 1st and 5th minutes were 9 and 10, respectively. Prenatal maternal laboratory studies were unknown, and no medication was used during pregnancy. She was the first child of unrelated parents from Afghanistan. There were no neurologic disorders, mental retardation, or hereditary disease in her family history. She was discharged from the hospital in good condition postnatal 24th hours of life. On the fifth day of life, a routine physical examination was recorded as normal at our neonatology follow-up clinic. At the age of nine days, she was brought to the emergency room due to poor sucking, abnormal eye movement, and intermittent cyanosis. Her actual weight was 2670 g, her body length was 50 cm, and her head circumference was 35 cm. Physical examination revealed mild central hypotonia, hypoactivity, lethargy, absent rooting reflex, sucking reflex, and symmetrically weak Moro reflex. After her admission to the NICU, right side tonic eye deviation and a cluster of abnormal ocular and facial movements accompanied by desaturation and tachycardia were noticed. In addition, persistent hiccup attacks with desaturations were observed, accompanied by abnormal ocular movements. Seizure activity was observed on amplitude EEG in the NICU. Phenobarbital was given intravenously as a 20 mg/kg loading dose, followed by a 5 mg/kg maintenance dose. However, there was no reduction in the frequency of seizures. The conventional EEG showed burst suppression periods and multifocal active epileptic discharge on the 11th day of life (Figure 1). Therefore, a levetiracetam (LEV) loading dose of 30 mg/kg/day two times and then midazolam infusions were added. The daily seizure burden dramatically decreased after triple anti-seizure therapy. Midazolam infusion was tapering for two days and stopped. Multifocal rare spike and sharp waves with no burst suppression periods were seen on repeated EEG under phenobarbital and LEV treatment.

**Figure 2:**
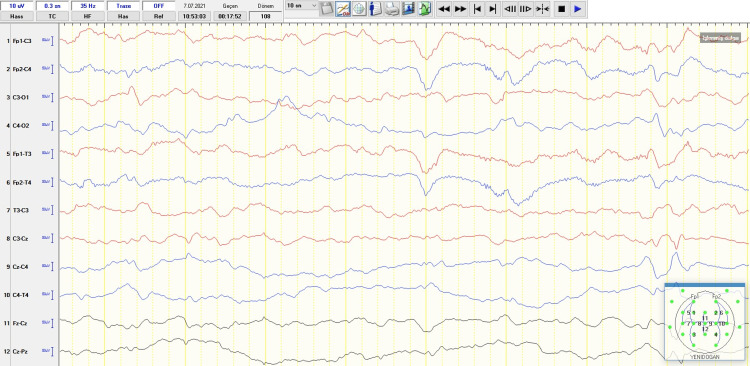
Normal background activity and rare sharp waves in central region were seen on EEG with newborn montage at 32 days of age.

Her first-line laboratory examination, including hemoglobin and platelet count, were 10.1 g/dL and 217x10^9^/L, respectively. Complete sepsis evaluation on admission was found negative for infection. The arterial blood gas analysis, blood glucose level, and serum electrolytes were within normal ranges, and she had a normal coagulation profile. TORCH (toxoplasmosis, rubella, cytomegalovirus, and herpes simplex virus) screen was negative, and the cell count, protein, glucose, and quantitative amino acids in cerebrospinal fluid (CSF) analysis were within normal limits. Serum lactate, pyruvate, ammonia, plasma and urine quantitative amino acids, tandem mass, urine organic acids, and very long-chain fatty acids were normal. CSF and plasma glycine levels (4.7 µmol/L and 200.28 µmol/L, respectively) and the ratio of CSF/plasma glycine (0.023) were normal, which ruled out non-ketotic hyperglycinemia. The blood sample was sent for the infantile epilepsy gene panel that includes IL6, IMPA2, KCNQ2, KPTN, MASS1/ADGRV1, MEF2C, NPRL2, NRXN1, NRXN2, PCDH19, PRRT2, PTGS2, and SCN1A. Cranial ultrasound demonstrated the variant of cavum septi pellucidi and cavum vergae, normal cerebral parenchymal sulcus, gyrus structure, and ventricular structure. Brain magnetic resonance imaging (MRI) was reported normal except for the variant of cavum septi pellucidi and cavum vergae. There was no clinical seizure activity under oral phenobarbital and LEV therapies in the NICU, and she was discharged with dual anti-seizure treatment on the 33^rd^ day of life. There was normal background activity and rare sharp waves in the central region without burst suppression pattern on EEG just before discharge (Figure 2). Phenobarbital was stopped at the first visit to the pediatric neurology outpatient clinic. Heterozygous c.1023G>C p.Gln341His mutation was detected in the KCNQ2 gene in the epilepsy gene panel. However, this mutation was not detected in the mother and father of the infant. She was diagnosed with EIEE associated with de-novo KCNQ2 mutation. The seizures started again after over two months of a seizure-free period and continued despite the LEV dose being increased by 50 mg/kg. Therefore, carbamazepine was started at the 4th month of life during the follow-up. At the last control, the patient was 1.5 years old with mild global developmental delay and had been followed for nine months without seizures with merely 15 mg/kg/day of carbamazepine.

## Discussion

Although more common presentations of KCNQ2-related phenotypes are SLFNE and EIEE, neonatal encephalopathy with non-epileptic myoclonus and isolated intellectual disability without epilepsy have also been described. In addition, KCNQ2 mutation can be related to developing epilepsy in childhood [11]. EIEE resulting from KCNQ2 gene mutation has genetic and phenotypic heterogeneity. Therefore, suspicion of a KCNQ2-related disorder is mainly based on familial history, clinical presentation, and EEG pattern [10,12,13]. The progression of neurodevelopmental problems continues even if the seizures are under control [13].

Kato et al. reported that a total of 239 EIEE cases, 51 of which were diagnosed with Ohtahara syndrome and 104 were diagnosed with West's syndrome, were analyzed, and the KCNQ2 mutation was detected in 10 patients (4.2%) [10]. They suggested that genetic testing for KCNQ2 should be considered in infants with EIEE [10]. Recently, KCNQ2 mutation was detected in 13 cases among 752 patients who underwent epilepsy gene panel test in the retrospective study of Kim et al. [12]. The most common initial seizure types were tonic seizures with or without autonomic symptoms. Myoclonic or clonic seizures were also noticed [12]. While the onset of seizures in 11 patients was between one and three days of life, the remaining two patients had the first seizure at the seventh and 40th days of life [12]. The present case had a tonic seizure with abnormal ocular symptoms and autonomic features like desaturation and tachycardia on the ninth day of life. In addition, non-ketotic hyperglycinemia was suspected due to persistent hiccup attacks in our patient, and the ratio of CSF/plasma glycine was double-checked for confirmation, and it was in the normal range. According to our knowledge, hiccups are not an expected finding in the semiology of seizure in KCNQ2-related EIEE and have not been previously described in the literature. On the other hand, a patient with KCNQ2-related seizures had intrauterine hiccups that the mother noticed in the last trimester of pregnancy, but no hiccups detected after birth as a seizure semiology have been reported [14].

Although autosomal dominant KCNQ2 mutation is traditionally associated with SLFNE, de-novo mutations are associated with EIEE [12]. Kato et al. described that 90% of patients with KCNQ2 mutation had de novo mutation, the remaining 10% had inherited mutation, and 80% of patients with KCNQ2 mutation became seizure-free with carbamazepine, zonisamide, phenytoin, topiramate, or valproic acid [10]. Although the seizures were relatively well controlled, moderate-to-profound intellectual disability was found in their series [10]. The main anti-epileptics that control seizures of the KCNQ2 cases in the study of Kim et al. were sodium channel blockers (11/13 cases, 85%) [12]. They found that sodium channel blockers such as oxcarbazepine (n=3), lamotrigine (n=3), phenytoin (n=2), topiramate (n=2), and zonisamide (n=1) were particularly effective [12]. Seizures of two patients were controlled with LEV without using a sodium channel blocker in their series of 13 patients. Seizures were controlled after the ketogenic diet was added to one and the other to valproic acid, pregabalin, and the ketogenic diet [12]. In the study of Kim et al., phenobarbital was seen as the most ineffective antiepileptic for EIEE patients with KCNQ2 mutation [12]. Phenobarbital and LEV were not successful in permanently controlling our patient's seizures.

We detected c.1023G>C p.Gln341His mutation in our patient. Only one case in the literature had the same KCNQ2 mutation, published in 2017 by Klotz et al. [13]. The seizures of their case ceased entirely after vitamin B6 was added to LEV and phenobarbital without using a sodium channel blocker [13]. In addition, Reid et al. reported a case of KCNQ2 channelopathy, suggesting that vitamin B6 may be a promising adjunctive treatment for patients with channelopathies and the wider epileptic population [14]. The seizures of their case were controlled with the combination of vitamin B6 and carbamazepine [14].

Recently, Kuersten et al. reported that 65.5% of patients with EIEE-related epilepsy were reported seizure-free [15]. In comparison, 14.3% had no treatment success in a systematic review (n=84). Of those who received monotherapy, 18.7% used sodium channel blockers, 14.5% used phenobarbital, and 16.6% used other anti-epileptics. The distribution of seizure medication of patients achieving seizure freedom was as follows: 37.8% out of all treatment trials with sodium channel blockers, 26.3% out of all treatment trials with valproic acid, and 33.3% out of all treatment trials with levetiracetam lead to seizure freedom [15]. Seizure control could not be achieved in 74% of the patients who received the initial phenobarbital treatment [15]. According to their series, 26 patients were treated with polytherapy, 20 became seizure-free, and zero showed no success at all [15]. More recently, Chen et al. reported that sodium channel blockers and other anti-epileptic drugs failed to cease seizures in four EIEE cases with KCNQ2 mutation [16]. The seizures of these cases ceased with vitamin B6 treatment. In addition, they suggested that pyridoxine may be a promising treatment for this group of patients [16]. Vitamin B6 was not started in the present patient because the seizures were under control with the treatment of the anti-seizure drugs before and after discharge.

## Conclusions

EIEE patients with KCNQ2 mutation is a very rare neurological disorder characterized by multidrug-resistant seizures. Although seizures in EIEE cases have variable semiology in the neonatal period, the most common initial seizure types were tonic seizures with or without autonomic symptoms. Persistent hiccups were described for the first time in the literature as part of the seizure semiology of KCNQ2-related EIEE. Sodium channel blockers have been reported as the most beneficial antiepileptic agents in multiple case series, and some case series suggested that pyridoxine was a promising adjunctive treatment.
